# A technique system for the measurement, reconstruction and character extraction of rice plant architecture

**DOI:** 10.1371/journal.pone.0177205

**Published:** 2017-05-30

**Authors:** Xumeng Li, Xiaohui Wang, Hailin Wei, Xinguang Zhu, Yulin Peng, Ming Li, Tao Li, Huang Huang

**Affiliations:** 1 Agricultural Mathematical Modeling and Data Processing Center, Hunan Agricultural University, Changsha, China; 2 International Rice Research Institute, Metro Manila, Philippines; 3 State Key Laboratory of Hybrid Rice, Changsha, China; 4 Hunan Agricultural University, Changsha, China; The National Orchid Conservation Center of China; The Orchid Conservation & Research Center of Shenzhen, CHINA

## Abstract

This study developed a technique system for the measurement, reconstruction, and trait extraction of rice canopy architectures, which have challenged functional–structural plant modeling for decades and have become the foundation of the design of ideo-plant architectures. The system uses the location-separation-measurement method (LSMM) for the collection of data on the canopy architecture and the analytic geometry method for the reconstruction and visualization of the three-dimensional (3D) digital architecture of the rice plant. It also uses the virtual clipping method for extracting the key traits of the canopy architecture such as the leaf area, inclination, and azimuth distribution in spatial coordinates. To establish the technique system, we developed (i) simple tools to measure the spatial position of the stem axis and azimuth of the leaf midrib and to capture images of tillers and leaves; (ii) computer software programs for extracting data on stem diameter, leaf nodes, and leaf midrib curves from the tiller images and data on leaf length, width, and shape from the leaf images; (iii) a database of digital architectures that stores the measured data and facilitates the reconstruction of the 3D visual architecture and the extraction of architectural traits; and (iv) computation algorithms for virtual clipping to stratify the rice canopy, to extend the stratified surface from the horizontal plane to a general curved surface (including a cylindrical surface), and to implement *in silico*. Each component of the technique system was quantitatively validated and visually compared to images, and the sensitivity of the virtual clipping algorithms was analyzed. This technique is inexpensive and accurate and provides high throughput for the measurement, reconstruction, and trait extraction of rice canopy architectures. The technique provides a more practical method of data collection to serve functional–structural plant models of rice and for the optimization of rice canopy types. Moreover, the technique can be easily adapted for other cereal crops such as wheat, which has numerous stems and leaves sheltering each other.

## Introduction

A plant architecture is defined by a set of features describing the shape, size, location, and orientation of the three-dimensional (3D) organization of the plant body above ground [1]. This architecture is of major agronomic importance because it strongly influences the adaptability of a crop for cultivation, dry matter production, and harvest indexing [1–2]. The architecture is also cultivar dependent and varies substantially among genotypes [3]. Moreover, the plant architecture can be modified by environmental factors, such as light, temperature, humidity, and nutrient status, which facilitate the dynamics of physiological processes such as stomata aperture, photosynthesis, respiration, nitrogen allocation, and photo morphogenesis [4]. With variations in plant architectures, changes in the microclimate of the plant canopy result in the adjustment of physiological processes [5–6]. Since the 1960s, functional–structural plant models have been developed to understand the relationship of 3D plant architectures with selected physiological processes [7–9].

Rice (*Oryza sativa* L.) is one of the dominant grain crops in most developing countries and is the staple food of more than half the world’s population [10]. In fact, the semi-dwarf rice varieties developed during the ‘green revolution’ in the 1960s possessed an improved plant architecture, an enhanced resistance to lodging caused by wind and heavy rain given their shorter stature, and better yield with higher harvest index [3]. In addition to efforts on breeding, the effects of various agronomic practices (e.g., water and fertilizer application, transplanting density, planting date and mulching mode) on rice architecture and yield, as well as the interaction between physiology and architecture, have also been investigated [11–13]. The 3D plant architecture of rice has been especially studied for the optimization of light distribution, photosynthesis, and yield in regards to cultivation management and breeding [14].

In the agricultural context, different types of sensor techniques, such as RGB cameras, 3D laser scanners, sonic and magnetic digitizers, and stereophotogrammetry, have been introduced [15]. In addition, many approaches have been developed to facilitate plant phenotypic research. 2D approaches based on 2D image processing and visible light imaging technology for leaf phenotypic analysis (to determine the leaf shape, size, width, length, area and perimeter), yield-related trait analysis (to determine the number of tillers, the panicle length and the grain size, length, width and thickness), and general plant analysis and measurement (to determine the plant height, plant width, center of gravity, projected area and biovolume) [16] have been developed. The 3D approaches based on 3D laser scanning techniques or sonic and magnetic digitizers, multisonic and magnetic digitizers, stereophotogrammetry have also been developed to reconstruct 3D plant architectures and extract plant traits [17–22]. However, many features cannot be extracted using 2D techniques (such as leaf area distributions and light distribution simulations), and equipment used in 3D approaches are too expensive. In particular, because rice planted densely in a field has numerous stems and leaves sheltering each other, previous approaches hardly apply to large-scale measurements and 3D architecture reconstruction for a whole plant.

The objectives of this study were, thus, to establish a low-cost, accurate and high-throughput approach to collect architectural data, to reconstruct the 3D visual architecture of a rice canopy, and to extract the architecture traits of a rice canopy.

## Materials and methods

### Plant material

Two experiments of this study were conducted in two agricultural research farm stations in Hunan Agricultural University at Liuyang, Hunan, China (113.51E, 28.23 N, 133.11 m elevation) and International Rice Research Institute in Los Baños, Philippines (21.25E, 14.18N, 21 m elevation) (check the details in S1 File). There was no permission needed to conduct an agricultural research in these two research farms because of their functional definition. The fields in both locations were managed with full water and nutrient supply. Except for the local popular rice cultivars, there were no endangered or protected species involved.

Seven groups of samples (S1 to S7) were collected from these two field experiments. Samples S1 and S2 were collected from 8 neighboring hills (two rows × four hills) for the reconstruction of the 3D visual canopy architecture, trait extraction, and the validation of light distribution within canopy. Sample S3 was collected from 4 neighboring hills for the validation of the leaf area distribution along the vertical direction (z-axis). Samples S4 and S5 were collected for the visual comparison between the reconstructed plant architecture and photos of the original architecture taken before the measurement of samples. Sample S6 was collected for the assessment of the stem position measurement. Sample S7 was collected for validating the calculation of the leaf length, maximum width, and area and for the reconstruction of the leaf orientation.

### General description of the technique system

**Fig 1:**
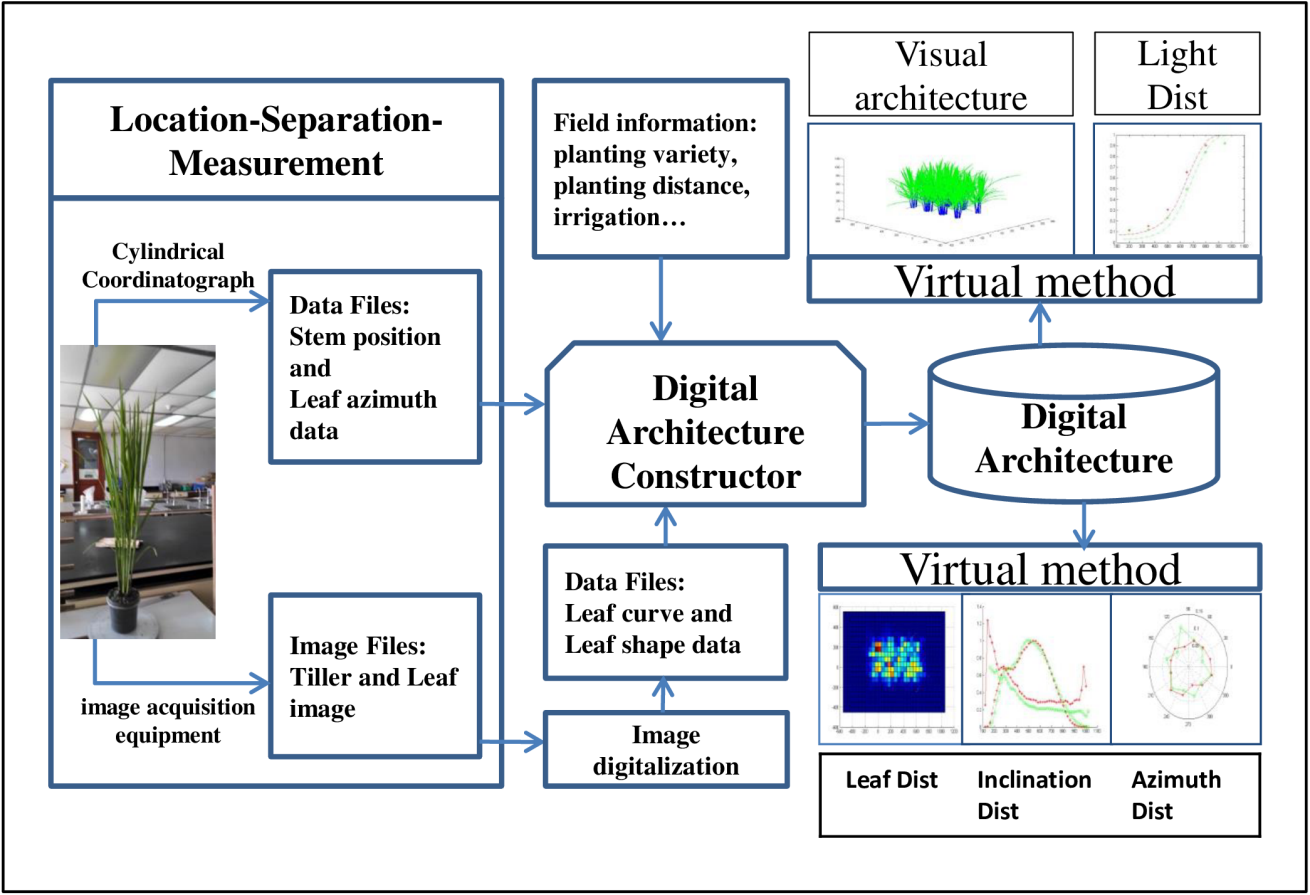
General schematic diagram of the technique system.

Aiming the establishment of the digital architecture in this study, the method, equipment, and software program for image capture and analysis were developed to collect the data for the digital architecture of rice plant (Fig 1). Data on the plant architecture were obtained based on the location-separation-measurement method (LSMM), and the spatial position and azimuth of a leaf were measured by a Cylindrical Coordinatograph (CC). Data on leaf shape and midrib curve were derived from photos of tillers and leaves captured by the image acquisition equipment. Both the reconstruction of the 3D virtual image of the canopy and the extraction of key traits (leaf area distribution, inclination and azimuth distribution, and light distribution) were performed using the developed computation algorithms based on a digital architecture.

### Measurement method and process

In this study, LSMM was developed to collect data on plant architectures. To measure the topological structure and geometric structure of an object consisting of sub-units, LSMM consists of three processes: (1) locating the position of the subunits, (2) separating the subunits from the object, and (3) measuring the topological structure or geometric structure of the sub-units. Data of plant architectures was collected for three types of objects: community (i.e. multiple hills), single hill, or single tiller of the rice plant, which was described in the details in S2 File.

**1.** Measurement of the plant architecture of a community. The location of a hill in the field was determined based on a row index, a column index, and north as a direction. With its anchor soil still attached, the hill was moved from the field to the laboratory, where the plant architecture was measured.

**2.** Measurement of the plant architecture of a single hill. The location of a tiller was determined by the stem spatial position and leaf azimuth using CC. To separate the tiller from the hill, the stem was cut at the soil surface. The radius of the stem and the curve of the leaf midrib of the tiller were derived by the software developed in this study using the tiller image taken by the tiller image acquisition equipment (TIAE).

**3.** Measurement of the topological structure and geometric structure of a single tiller. The location of a leaf was determined by the curve of the leaf midrib and leaf azimuth. The leaf was separated from the stem at the leaf node. The leaf shape was determined by the developed software using the leaf image captured by the leaf image acquisition equipment (LIAE).

For a multiple hill community, the LSMM was described in the details in S2 File.

### Image analysis

For extracting the leaf midrib curve and leaf shape from the tiller and leaf images, computing software that includes image binary, image dilation and erosion, image label, and image rotation was developed in MATLAB (2009a).

#### Leaf image analysis

The leaf shape profile data can be extracted through leaf image analysis with the following steps (see the steps in S1 Fig).

**1.** The real coordinates of each pixel were transformed from image pixel column and row numbers using a group of binary quadratic functions (Eq 1),

$$\left\{ \begin{array}{c} x=a_{1}∙i^{2}+b_{1}∙i∙j+c_{1}∙j^{2}+d_{1}∙i+e_{1}∙j+f_{1}\\ y=a_{2}∙i^{2}+b_{2}∙i∙j+c_{2}∙j^{2}+d_{2}∙i+e_{2}∙j+f_{2} \end{array}\right.$$ (1)

where *i* and *j* are the pixel column and row, *x* and *y* are the coordinates in real numbers, and *a*_*1*_, *b*_*1*_, *c*_*1*_, *d*_*1*_, *e*_*1*_, *f*_*1*,_ *a*_*2*,_ *b*_*2*_, *c*_*2*_, *d*_*2*_, *e*_*2*_, and *f*_*2*_ are the function parameters determined by the least-squares criterion to minimize the root mean square error between estimated distances among neighboring points from validation paper image (S2 File) and actual measured distance.

**2.** A binary image was generated using three processes: gray, binary, and filter. The graying function (Eq 2) was used to obtain the gray value (Gray(*i*,*j*)) of point (*i*,*j*),

Gray(i,j)=G(i,j)−R(i,j)/3−B(i,j)/3 (2)

where G(*i*,*j*) is the green value, R(*i*,*j*) is the red value, and B(*i*,*j*) is the blue value of point (*i*,*j*) in a color image. Gray image was converted to binary image based on global image threshold computed by Otsu’s method. Binary image was filtered using [3, 3] average filter.

**3.** For a given leaf, its dataset of real coordinates (*L*) for defining the leaf shape was calculated by Eq 1 after the leaf was isolated from the binary image.

**4.** The leaf width along the leaf vein was derived using Eq 3 after the binary image was processed. First, the leaf coordinate was rotated to parallel the leaf vein approximately with the abscissa axis and was also shifted at the center of the leaf bottom to the coordinates (0,0). In Eq 3, for a point (*x*,*y*) on the leaf in (*x*,*y*)*∈L*, *L*_*w*_*(L*_*l*_*)* is the leaf width at this point, where the horizontal distance to the leaf node is *L*_*l*_ and the *L*_*mw*_ is the maximum leaf width. For a given leaf *L*, the leaf length is the maximum *L*_*l*_, and the leaf width changes from leaf node to leaf tip, defined as Eq 3,

$$L_{w}\left(L_{l}\right)=\left\{\max\left(y\right)-\min\left(y\right)|\left(L_{l},y\right),\left(x,y\right)\in L\right\}$$ (3)

where ε is a sufficiently small positive real number.

**5.** The leaf shape curve function as a 6^th^-degreepolynomial was derived from the data of *L*_*l*_ and *L*_*W*_ (*L*_*l*_) /2 (Eq 4),

$$L_{w}\left(L_{l}\right)=aL_{l}{}^{6}+bL_{l}{}^{5}+cL_{l}{}^{4}+dL_{l}{}^{3}+eL_{l}{}^{2}+fL_{l}{}^{1}+g.$$ (4)

where *a*, *b*, *c*, *d*, *e* and *f* are the function parameters and *g* is the residual of the function.

#### Tiller image analysis

The leaf venation profile data were extracted from the tiller image through the following analysis (see the analysis step in S2 Fig).

**1.** The real coordinates of each pixel were transformed from image pixel column and row numbers using a group of binary quadratic functions (Eq 1).

**2.** A binary image of the tiller was generated with the partition method through binary and filter processing, in which the variation in light intensity in the larger area of the tiller span was reduced. After gray processing, the image was partitioned into 16 equal quadrate sub-blocks, on which binary processing were conducted same as leaf image analysis, filtering by [2, 2] average filter.

**3.** The stem and leaves were separated from the binary image by extracting the stem at the leaf node according to the character of the topological structure, splitting the leaves into separate parts and labeling each organ (i.e. leaf and stem).

**4.** The dataset of the real coordinates of the stem for defining the stem shape (*S*) and the dataset of the real coordinates of the leaves for defining the leaf curve (*Lc*) were calculated by Eq 1 after the stem and leaves were isolated from the binary image.

**5.** To determine the length and radius of the stem (*S*_*l*_, *S*_*r*_) as well as the position of the leaf node and the point set of the midrib of a leaf (LN, LM), the coordinates were rotated to parallel the stem approximately along the vertical axis and were also shifted at the center of the stem bottom to the coordinates (0, 0). For a given stem *S*, its length and radius at different positions from bottom to top were derived using Eqs 5 and 6. For a given leaf *Lc*, its node position as the attached point of the leaf on the stem and the point dataset of the midrib of the leaf were derived using Eqs 7 and 8, where *Sr*(*z*) is the stem radius at a distance *z* from the stem bottom.

$$S_{l}=\{\max\left(v\right)|(u,v)\in S\}$$ (5)

$$S_{r}(z)=\{max(x)||u-x|<\varepsilon ,(u,z)\in S\}$$ (6)

LN={(x,y)|x=min(|u|),y=mean(z),|w−x|<ε,(w,z),(u,v)∈Lc} (7)

LM={(x,y)|x=u,y=mean(z),|w−x|<ε,(w,z),(u,v)∈Lc} (8)

**6.** The leaf midrib curve was described by the parameters *x*_*lm*_(*l*) and *y*_*lm*_(*l*) (Eq 10), which fit the point dataset of the midrib (*LMO*, Eq 9) in reference to the coordinate system, with the origin at the leaf node and the longitudinal axis straight up, where *l* is the leaf length from *(x*,*y)* to the leaf node.

LMO={(x−u,y−v)|(x,y)∈LM,(u,v)=LN}. (9)

$$\left\{ \begin{array}{l} x_{lm}(l)=a_{1}l^{2}+b_{1}l+c_{1}\\ y_{lm}(l)=a_{2}l^{2}+b_{2}l+c_{2} \end{array}\right.$$ (10)

### Digital plant architecture

In this study, the digital plant architecture was defined using structural data with spatial position reference, shape, and size for different objects, from hills in the field to the stem and leaves attached to the plant, and using operations for the reconstruction of the 3D virtual plant architecture and trait extraction. The structural data are defined as described in S2 File:

Structural data were collected through experimental recording, measurement, and image analysis. In this study, the structural data of the digital geometrical structure were obtained in a 3D Cartesian Coordination System that corresponds to the cylindrical coordinate system, with the orientation along the horizontal plane, the longitudinal axis at the center of the stems, and the polar axis given by the marked azimuth of the hill.

The planting space is the row and plant space represented as (*rs*, *ps*).

The hill position in the field is represented by the row and column numbers in the sampling block as (*rn*, *cn*).

The stem length is denoted by *S*_*l*_ calculated by Eq 5, and the stem spatial position is represented by two endpoints (*x*_*b*_, *y*_*b*_, *0*) and (*x*_*t*_, *y*_*t*_, *z*_*t*_) in Eq 11, where j = 1,2 and (*r*_*j*_, *a*_*j*_, *h*_*j*_) is a point of the stem measured by the Clindrical Coordinatograph method described in S2 File.

$$\left\{ \begin{array}{l} \frac{h_{2}-h_{1}}{h_{1}-0}=\frac{x_{2}-x_{1}}{x_{1}-x_{b}}=\frac{y_{2}-y_{1}}{y_{1}-y_{b}},\\ \frac{x_{t}-x_{b}}{x_{1}-x_{b}}=\frac{y_{t}-y_{b}}{y_{1}-y_{b}}=\frac{z_{t}-z_{b}}{z_{1}-0}=\frac{Sl}{\sqrt{{\left(x_{1}-x_{b}\right)}^{2}+{\left(y_{1}-y_{b}\right)}^{2}+{\left(h_{1}-0\right)}^{2}}}\\ x_{j}=r_{j∙}cos(a_{j}),y_{j}=r_{j∙}sin(a_{j}) \end{array}\right.$$ (11)

The stem radius is denoted by *SR(z*_*i*_*)*, *i = 1…N*, *and z*_*j-1*_*<z*_*j*_, *j = 2…N* obtained through Eq 6. The leaf azimuth is denoted by *θ* measured by the Clindrical Coordinatograph method. The leaf node is denoted by (*x*_*ln*_, *y*_*ln*_,*z*_*ln*_) (Eq 12), where (*x*,*y*)*∈LN* is defined in Eq 7 and *z*_*t*_ is defined in Eq 11.

$$\frac{y}{z_{t}}=\frac{x_{ln}-x∙\cos\left(\theta \right)-x_{b}}{x_{t}-x_{b}}=\frac{y_{ln}-x∙\sin\left(\theta \right)-y_{b}}{y_{t}-y_{b}}=\frac{z_{ln}}{z_{t}}$$ (12)

The leaf width is denoted by (*l*_i_,L_w_(*l*_i_)) in Eqs 3 and 4. The leaf midrib curve is denoted by (*l*_*i*_, *x*_*lm*_,(*l*_i_) *y*_*lm*_(*l*_i_) *z*_*lm*_(*l*_i_)), *l*_*i-1*_*<l*_i_, *i = 1…N*, as Eq 13, where (*l*_*i*_, *x*_*lm*_,(*l*_i_) *y*_*lm*_(*l*_i_)) is calculated from Eq 10, and *S*_*L*_ is the stem length as in Eq 11:

$$\left\{ \begin{array}{l} x_{lm}(l_{i})=x_{lm}(l_{i})∙\cos\left(\theta \right)+x_{ln}\\ y_{lm}(l_{i})=x_{lm}(l_{i})∙\sin\left(\theta \right)+y_{ln}\\ z_{lm}(l_{i})=y_{lm}(l_{i})∙\frac{zt}{SL} \end{array}\right.$$ (13)

The leaf shape is denoted by (*l*_*i*_, *x*_*ls*_,(*l*_i_) *y*_*ls*_(*l*_i_) *z*_*ls*_(*l*_i_)), *l*_*i-1*_*<l*_i_, *i = 1…N*, (*l*_*i*_, *x*_*ls*_,(*l*_i_) *y*_*ls*_(*l*_i_) *z*_*ls*_(*l*_i_)) are the spatial 3D coordinates of points on the leaf on two edges (Eq 14 and), where *P =* (*x*_*lm*_,(*l*_*m*_)- *x*_*lm*_,(*l*_*1*_), *y*_*lm*_(*l*_*m*_)- *y*_*lm*_(*l*_*1*_) *z*_*lm*_(*l*_*m*_)- *z*_*lm*_(*l*_*1*_)) × (*x*_*lm*_,(*l*_*N*_)- *x*_*lm*_,(*l*_*m*_), *y*_*lm*_(*l*_*N*_)- *y*_*lm*_(*l*_*m*_) *z*_*lm*_(*l*_*N*_)- *z*_*lm*_(*l*_*m*_)), P = (*p*_*1*_, *p*_*2*_, *p*_*3*_) is the vector perpendicular to the plane of leaf venation, *norm*(*P*) is the modulus function, the two terms on the two sides of the multiplication sign (×) are the two vectors defining a surface, and *P* is the productive vector defined by the vector components *p*_*1*_, *p*_*2*_ and *p*_*3*_.

$$\left\{ \begin{array}{l} x_{ls}(l_{i})=x_{lm}(l_{i})\mp \frac{L_{w}(l_{i})}{norm(P)}∙p_{1}\\ y_{ls}(l_{i})=y_{lm}(l_{i})\mp \frac{L_{w}(l_{i})}{norm(P)}∙p_{2}\\ z_{ls}(l_{i})=z_{lm}(l_{i})\mp \frac{L_{w}(l_{i})}{norm(P)}∙p_{3} \end{array}\right.$$ (14)

The data obtained from the above processes were further analyzed following these processes to address our research objectives: visualization of geometrical structure and the virtual clipping method.

### Reconstruction of the 3D virtual plant architecture

**Fig 2:**
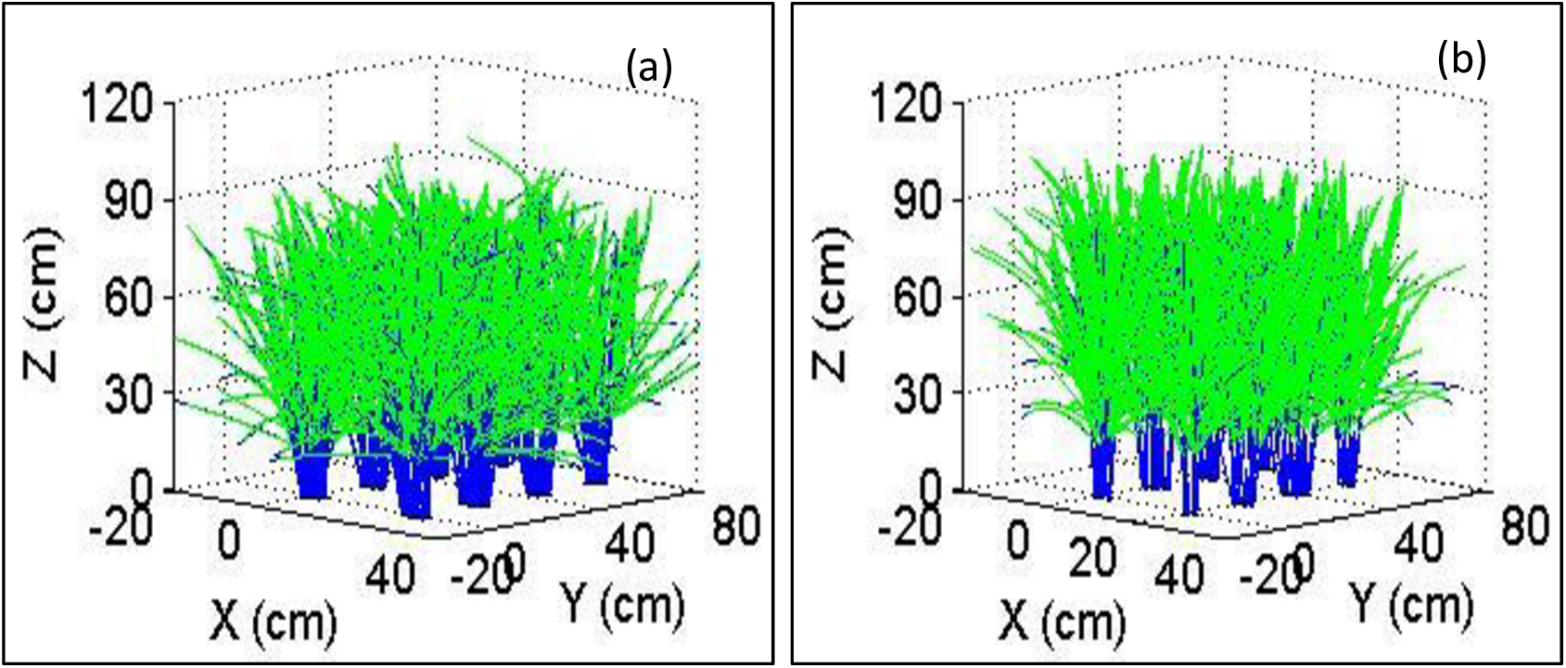
The 3D visual canopy architecture of rice in a field with eight hills. The (**a**) corresponds to Sample S1 (NSICRc222), and (**b**) corresponds to Sample S2 (NSICRc124H).

The 3D visual architecture of a plant in the field was reconstructed for the visualization of the geometrical structure (Fig 2). The stem was represented by a cylinder, and the leaves were represented by a wireframe surface. The visualization of the geometrical structure, according to the definition of the digital geometrical structure, is described in S2 File.

### Extraction of architecture traits using the virtual blade method

The stratified clipping method, which stratifies the canopy along a vertical direction into several layers and extra parameters of the leaf area and orientation in each layer, is extensively used to understand the relationship between light environment and biomass product and then to design a productive structure of the plant community. The virtual stratified clipping method, which combines the stratified clipping method with a virtual plant, was used to collect data on leaf area and orientation for each layer in the canopy -[18]. However, both clipping methods can only provide canopy information in a vertical space and not in a horizontal space. To extract information in both vertical and horizontal spaces, this study improved the virtual stratified clipping method and transformed it into a virtual blade method.

#### Concept of virtual blade method

The virtual blade method (S3 Fig) can be described by a series of mathematical equations (Eqs 15, 16 and 17). Two parallel spatial surfaces, A and B, called virtual blade surfaces, were defined by Eq 15, where *F*_B_(*x,y,z*)<*F*_A_(*x,y,z*) for any (*x,y,z*)*∈*R^3^, and *R^3^* is the 3D real space.

$$F_{A}(x,y,z)=0,\;F_{B}(x,y,z)=0$$ (15)

If a leaf surface can be described by Eq 16, the leaf-cut section determined by blade surfaces A and B can be written as Eq 17, where *i* is the series number of leaves and *Ω = ⋃Ω*_*i*_ is the integration of all leaf-cutsections of all leaves. Thus, traits between sections A and B can be computed in Ω.

$$F_{i}(x,y,z)=0\;i=1\ldots n$$ (16)

$$\Omega_{i}=\{(x,y,x)|F_{A}(x,y,z)>0,F_{B}(x,y,z)<0,F_{i}(x,y,z)=0\}$$ (17)

Various virtual blade sections can be chosen for a specific objective. In the case of horizontally homogeneous canopies, if a set of horizontal sections can be chosen as virtual blade sections to investigate the density function along the vertical axis, the virtual blade technology is equivalent to the virtual stratified clipping method. In the case of concentric circle homogeneous canopies, such as *sago cycas*, a set of cylindrical surfaces are chosen as virtual blade sections to investigate the leaf areadensity function on the distance from the central vertical axis of a hill (i.e. hill axis). For the rice canopy, where the leaf azimuth is supposed to be homogeneous, the cylindrical surface virtual blade can also be used for the density function on the distance from the hill axis.

#### Virtual blade algorithm

However, it is difficult to determine the trait distribution using the virtual blade method directly because we have to solve many equations for the intersections of surfaces. To simplify the virtual blade method, we proposed the use of algorithms for the virtual blade method (virtual blade algorithms) to compute an approximately trait distribution. Based on the 3D digital geometrical structure, the virtual blade algorithm for extracting leaf trait distributions is described by the following steps:

1. Reconstruct the 3D digital geometrical structure;
2. Define a set of parallel blade sections, *F*_*k*_(*x*,*y*,*z*) = 0, *k = 1*,*2…*,*m* and a set of interspace {(*x*,*y*,*x*)|*F*_*A*_(*x*,*y*,*z*)>0,*F*_*B*_(*x*,*y*,*z*)<0,*F*_k_(*x*,*y*,*z*) = 0}, *k = 1*,*2⋯m-1;*
3. Divide the leaves into fragments and compute the traits of each fragment;
4. Summarize the traits of each fragment in intervals between parallel blade sections *F*_*k*_(*x*,*y*,*z*) = 0 and *F*_*k+1*_(*x*,*y*,*z*) = 0. The details of the algorithms are presented in S2 File.

#### Virtual blade for multi-dimension distribution of architecture traits

If the difference in architecture traits at different positions in a flat space or 3D space is investigated, virtual blade algorithms can be adopted for 2D or 3D distributions of architecture traits via the extension of a single-group blade to a multi-group blade. Three types of planes parallel to the *xoy*, *xoz*, and *yoz* planes can often be chosen as virtual blades. Sequentially, the space of the canopy is split into *m×n* or *m×n×l* sub-spaces named voxels. The process for generating a virtual blade is described in S2 File:

### Spatial distribution of leaf area

#### The leaf area distribution along the vertical direction (z-axis)

**Fig 3:**
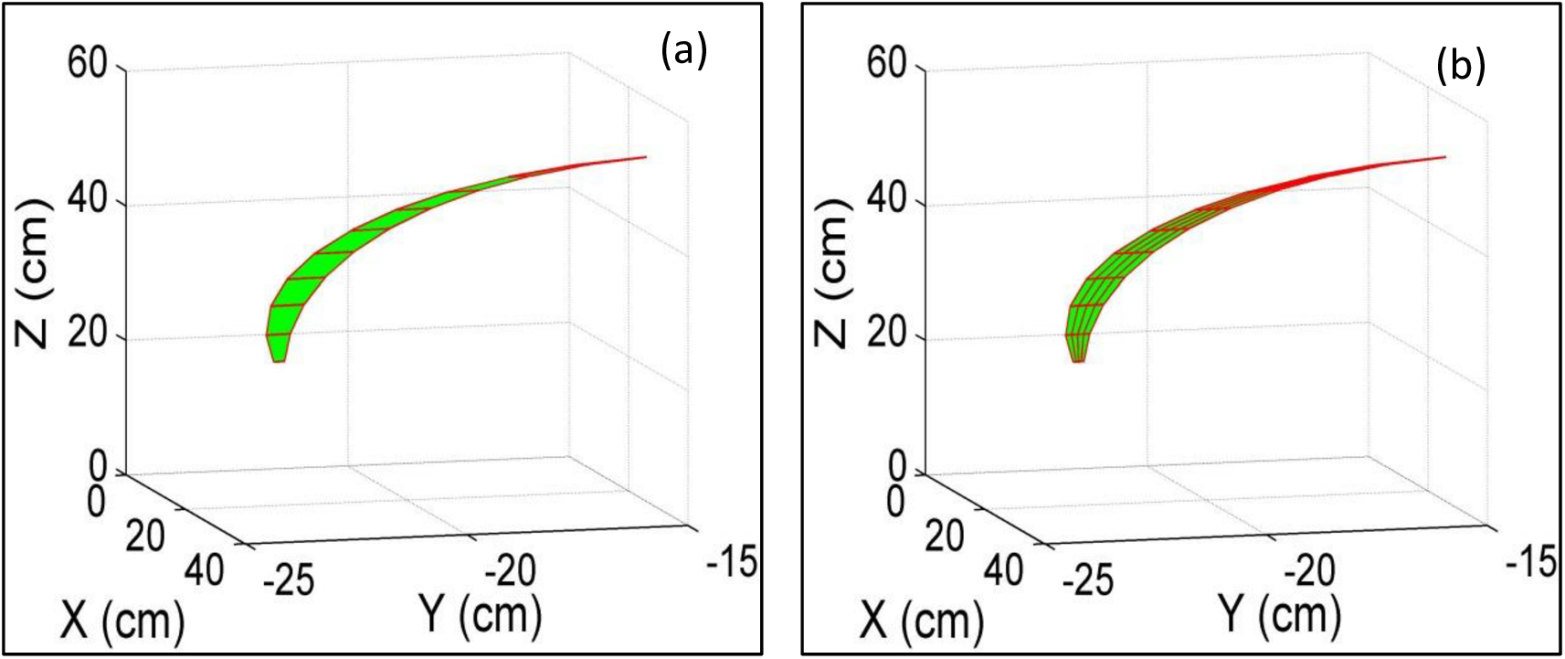
Schematic diagram of dividing a special leaf into fragments.

The leaf area density along the z-axis was investigated using the virtual blade method (S2 File). In this process, horizontal surfaces were chosen as virtual sections, and the distance between two adjacent surfaces was *D_ns_* (cm) (see S3 Fig); the leaf was divided into a number of fragments across the leaf vein (Fig 3A). The order number of intervals for the fragments was calculated by Eq 18, where *floor*(*x*) rounds *x* to the nearest integer less than or equal to *x*.

$$finterval=floor\left(\frac{z_{lm}(l_{i})+z_{lm}(l_{i+1})}{{2*D}_{ns}}\right)+1.$$ (18)

The area of a fragment as an experimental trait was also calculated by Eq 19, where *l*_*i+1*_ and *l*_*i*_ (cm) are the curve distances on the leaf vein from the upper and lower ends of the *i*^*th*^ fragment to the leaf node, and *L*_w_(l_i+1_) and *L*_w_(*l*_i_) are the leaf width at the upper and lower sides of the *i*^*th*^ fragment.

$${farea=(l}_{i+1}-l_{i})∙(L_{w}{(l}_{i+1})+L_{w}{(l}_{i}))/2$$ (19)

The leaf area in interval *n* was determined by Eq 20, the leaf area density along the z-axis was determined by Eq 21, and the leaf area probability density along the z-axis was determined by Eq 22.

$$Area(n)=\sum_{finterval==n}farea$$ (20)

$$Areadis(n)=Area(n)/D_{ns}/pm/rs/ps$$ (21)

Areapdis(n)=Areadis(n)/∑Area(n) (22)

**Fig 4:**
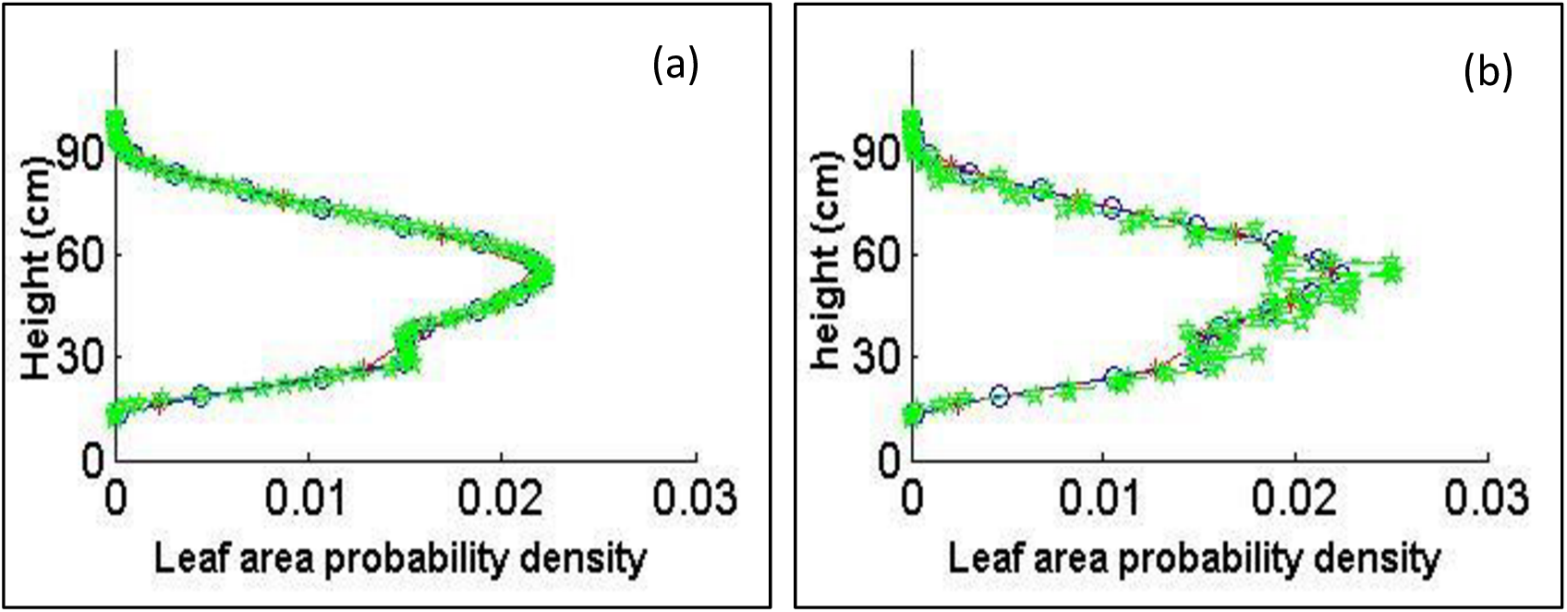
**Leaf area distribution along the z-axis in (a) with fragment number *fnum = 100* and (b) with fragment number *fnum = 10***. The red lines are for *D*_*ns1*_ *= 10* cm, the blue lines are for *D*_*ns2*_ *= 5* cm, and the green lines are for *D*_*ns3*_ *= 1* cm.

The distances between two neighboring sections (see S3 Fig) were chosen as *D*_*ns1*_ *= 10* cm, *D*_*ns2*_ *= 5* cm, and *D*_*vn3*_ *= 1* cm, and the leaves were divided into *fnum* fragments (*fnum = 10*, *fnum = 100*). The leaf area density along the z-axis is presented in Fig 4.

When *fnum = 100*, the leaf area density along the z-axis was different among *D*_*ns*1_ *= 10* cm, *D*_*ns*2_ *= 5* cm, and *D*_*vn*3_ *= 1* cm at z-axis intervals from 26 to 38 cm and from 47 to 64 cm (Fig 4A), which implied that the small distance between neighboring sections was helpful in accurately representing the leaf area distribution. When *fnum = 10* and *D*_*ns*3_ *= 1* cm, the leaf area distribution varied substantially (green line in Fig 4B), which means that the distribution was described statistically with large leaf fragments. In this case, *fnum = 100* and *D*_*ns2*_ *= 5* cm could be the most appropriate choice for the accurate description of the leaf area distribution.

#### Area distribution as a function of the distance to the hill axis

The leaf area density as a function of the distance to the hill axis was investigated using the virtual blade method (S3 Fig). Cylinder surfaces surrounding the hill axis were chosen as the virtual section, and the leaves were divided into *fnum* fragments along the leaf midrib (Fig 3A).

The area of the fragment was calculated using Eq 19, and the interval of the fragments was calculated using Eq 23.

$$finterval=floor(\sqrt{{\left(x_{lm}(l_{i+1})+x_{lm}(l_{i})\right)}^{2}+{\left(y_{lm}(l_{i+1})+y_{lm}(l_{i})\right)}^{2}+{\left(z_{lm}(l_{i+1})+z_{lm}(l_{i})\right)}^{2}}/D_{ns})+1$$ (23)

The area in interval *n* is determined using Eq 20, and the leaf area density distribution as a function of the distance to the hill axis is determined by Eq 24.

$$Areadis(n)=Area(n)/\pi ({\left(nD_{ns}\right)}^{2}-{\left((n-1)D_{ns}\right)}^{2})/pm$$ (24)

**Fig 5:**
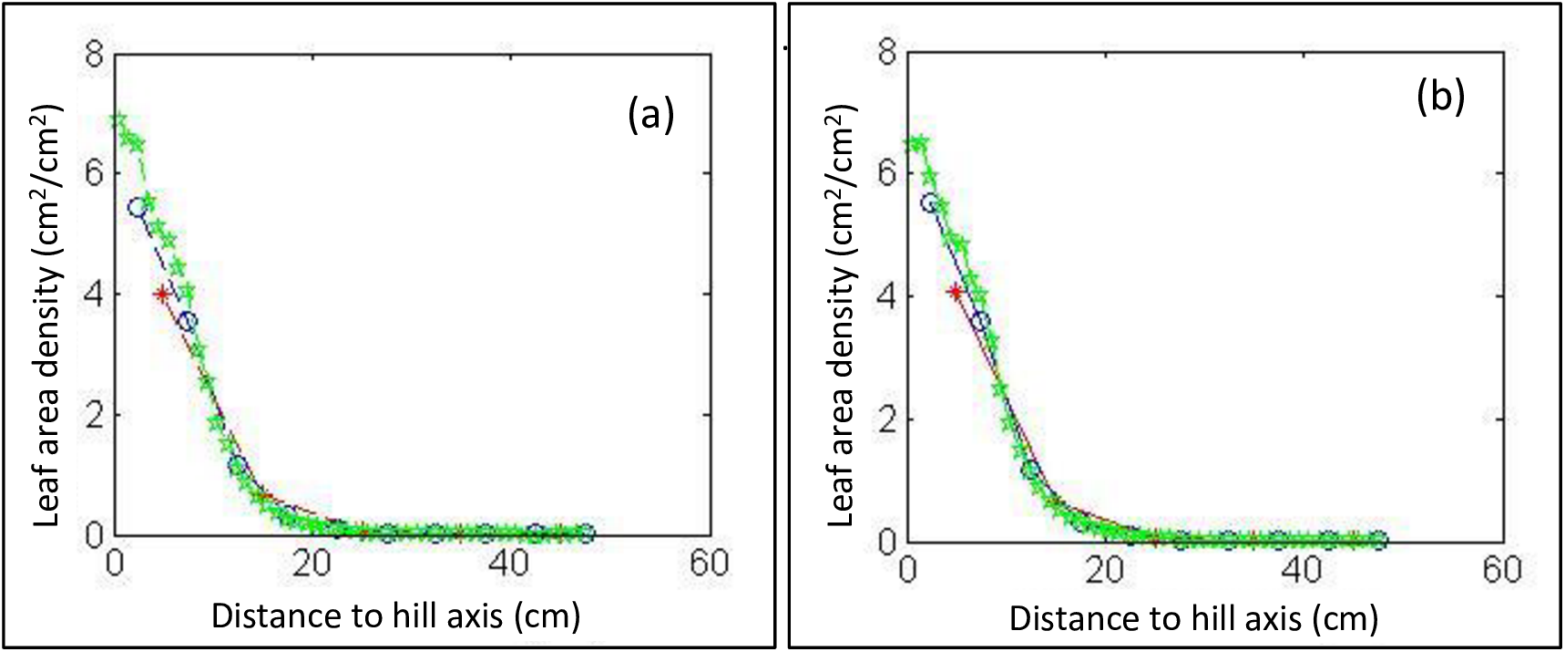
**Leaf area distribution as a function of the distance from the axis of a hill in (a) with fragment number *fnum* = 100 and (b with fragment number *fnum = 10***. The red lines represent *D*_*ns1*_ *= 10* cm, the blue lines represent *D*_*ns2*_ *= 5* cm, and the green lines represent *D*_*ns3*_ = 1 cm.

The distance between two neighboring cylinder surfaces was tested under three assumptions: *D*_*ns*1_ *= 10* cm, *D*_*ns2*_ *= 5* cm, and *D*_*ns3*_ *= 1* cm, with two leaf fragment numbers (*fnum = 10*, *fnum = 100*). The area density distribution as a function of the distance to the hill axis is shown in Fig 5, which illustrates that the leaf area distribution was affected significantly by distance between adjacent virtual surfaces, but not significantly by the fragment number.

#### Leaf area density on the xyz (or xoy) plane in a multiple-hill community

The leaf area density on the XYZ (or XOY) plane was investigated using the algorithm for virtual point vertical quadrate sampling (S2 File), where Z is the vertical axis of hill, while the X and Y are the axes for the directions between rows and between plants. The *xyz* plane was divided into voxels with a distance along the x-axis (*D*_*x*_), along the y-axis (*D*_*y*_), and along the z-axis (*D*_*z*_) and with each leaf divided into *fnum* fragments along the leaf midrib. Each fragment was represented by four points: (*l*_*i*_, *x*_*ls1*_,(*l*_*i*_) *y*_*ls1*_(*l*_*i*_) *z*_*ls1*_(*l*_*i*_)); (*l*_*i*_, *x*_*ls2*_,(*l*_*i*_) *y*_*ls2*_(*l*_*i*_) *z*_*ls2*_(*l*_*i*_)); (*l*_*i+1*_, *x*_*ls1*_,(*l*_*i+1*_) *y*_*ls1*_(*l*_*i+1*_) *z*_*ls1*_(*l*_*i+1*_)) and (*l*_*i+1*_, *x*_*ls2*_, (*l*_*i+1*_) *y*_*ls2*_(*l*_*i+1*_) *z*_*ls2*_(*l*_*i+1*_)).

Each first order of leaf fragment was furtherly divided into four secondary smaller fragments by the midrib and the two midlines of the midrib and edge (Fig 3B).

In this case, the virtual blade method algorithm was used, with the leaf area as the trait. The area of the fragment was calculated as *farea* = (*l*_*i+1*_-*l*_i_)(*L*_*w*_(*l*_*i+1*_)+*L*_*w*_(*l*_*i*_))/8. The voxels containing the fragments were determined by Eq 25. The leaf area in voxels (*n*_*x*_,*n*_*y*_,*n*_*z*_) was calculated by Eq 26, and the leaf area distribution on the *xoy* plane was calculated using Eq 27.

$$\left\{ \begin{array}{l} N_{x}=floor\left(\frac{2i-1}{8}x_{ls1}(l_{i})+\frac{8-2i}{8}x_{ls1}(l_{i+1})/D_{x}\right)+1\\ N_{y}=floor\left(\frac{2i-1}{8}y_{ls1}(l_{i})+\frac{8-2i}{8}y_{ls1}(l_{i+1})/D_{y}\right)+1\\ N_{z}=finterval=floor\left(\frac{z_{lm}(l_{i})+z_{lm}(l_{i+1})}{{2*D}_{ns}}\right)+1 \end{array}\right.$$ (25)

$$Area\left(n_{x},n_{y},n_{z}\right)=\sum_{N_{x}=n_{x},N_{y}=n_{y},N_{z}=n_{z}}farea$$ (26)

$$Areadis(n_{x},n_{y},n_{z})=Area(n_{x},n_{y},n_{z})/(D_{x}\times D_{y}\times D_{z})$$ (27)

### Spatial distribution of leaf orientation

#### Leaf azimuth distribution

The leaf azimuth distribution was investigated using the virtual blade method algorithm (S2 File). The 0 to 360 degree azimuth was divided into *dn* equivalent portions, and each leaf was divided into a number of fragments along the leaf midrib (*fnum*) (Fig 3A). The area of a fragment was calculated using Eq 19. The azimuth of a fragment was calculated by Eq 28, where (*l*_*i*_, *x*_*lm*_,(*l*_i_) *y*_*lm*_(*l*_i_) *z*_*lm*_(*l*_i_)) is the leaf midrib.

$$\begin{array}{l} fazimuth=\left\{ \begin{array}{l} \;\beta \;y_{lm}\left(l_{i+1}\right)-y_{lm}\left(l_{i}\right)>0;x_{lm}(l_{i+1})-x_{lm}(l_{i})>0\\ \;\pi -\beta \;y_{lm}\left(l_{i+1}\right)-y_{lm}\left(l_{i}\right)>0;x_{lm}(l_{i+1})-x_{lm}(l_{i})<0\\ \;\pi +\beta ,\;y_{lm}\left(l_{i+1}\right)-y_{lm}\left(l_{i}\right)<0;x_{lm}(l_{i+1})-x_{lm}(l_{i})<0\\ 2\pi -\beta \;y_{lm}\left(l_{i+1}\right)-y_{lm}\left(l_{i}\right)<0;x_{lm}(l_{i+1})-x_{lm}(l_{i})>0 \end{array}\right.\\ \beta =argtan\left(\left|\frac{y_{lm}(l_{i+1})-y_{lm}(l_{i})}{x_{lm}(l_{i+1})-x_{lm}(l_{i})}\right|\right) \end{array}$$ (28)

The interval in which the smaller fragments were located was calculated using Eq 29, the area of fragments in interval *n* was calculated using Eq 20, and the leaf azimuth distribution was calculated using Eq 30.

$$finterval=floor\left(\frac{fazimuth}{2\pi }dn\right)+1.0$$ (29)

Azimuthdis(n)=Area(n)/∑Area(i) (30)

#### Leaf inclination distribution along the z-axis

The leaf inclination distribution along the z-axis was investigated using the virtual blade method algorithm (S2 File), similar to the approach used for the leaf area density along the z-axis. The trait of the fragments (*farea*) was calculated using Eq 19, while the inclination was calculated using Eq 31. The fragments in interval *n* were calculated using Eq 18, and the leaf inclination distribution along the z-axis was calculated using Eq 32.

$$finclin=arcsin\frac{\left|z_{lm}(l_{i+1})-z_{lm}(l_{i})\right|}{\sqrt{{\left(x_{lm}(l_{i+1})-x_{lm}(l_{i})\right)}^{2}+{{\left(y_{lm}(l_{i+1})-y_{lm}(l_{i})\right)}^{2}+(z_{lm}(l_{i+1})-z_{lm}(l_{i}))}^{2}}}.$$ (31)

$$inclinationdis(n)=\sum_{intervalfrag=n}farea*finclin/\sum_{finterval=n}farea$$ (32)

#### Leaf inclination distribution as a function of the distance to the axis of a hill

The leaf inclination distribution as a function of the distance to the axis of a hill was investigated using the virtual clipping method (S2 File), similar to the approach used for the leaf area density as a function of the distance to the axis of a hill. The interval of the fragments was calculated using Eq 23. The traits of the fragments *farea* were calculated using Eq 19, and *inclinfrag* was calculated using Eq 18. The leaf angle distribution as a function ofthe distance to the axis of a hill was determined using Eq 32.

#### Light distribution along the z-axis

The light distribution along the z-axis was investigated using the virtual blade method algorithm (S2 File), similar to the approach used for the leaf area density function along the z-axis. The projection area on the plane perpendicular to the sun was calculated using Eq 33, where *θ*_*leaf*_ = *finclin*, *φ*_*leaf*_ = *fazimuth*, *θ*_*sun*_ is the solar altitude angles, and *φ*_*sun*_ is the sun azimuth angle.

$$\begin{array}{l} fPSAREA=farea*A(\theta_{sun},\theta_{leaf},\varphi_{sun},\varphi_{leaf})\\ A(\theta_{sun},\theta_{leaf},\varphi_{sun},\varphi_{leaf})=cos(\theta_{sun})cos(\theta_{leaf})+sin(\theta_{sun})sin(\theta_{leaf})cos(\varphi_{sun}-\varphi_{leaf}) \end{array}$$ (33)

The total projection area on the plane perpendicular to the sun by the fragments in interval *n* was calculated using Eq 34. The light interception coefficient of the interval *n* was calculated using Eq 35, and the light relative density was determined using Eq 36 [23], where PAR0 is the solar radiation on the upper portion of the canopy.

$$PSAREA\left(\text{n}\right)=\sum_{intervalfrag=n}\;fPSAREA$$ (34)

$$\text{LIC}\left(\text{n}\right)=1-\sum_{intervalfrag=n}fPSAREA/\text{plantnum}/\text{rows}/\text{cols}$$ (35)

$$\text{LID}\left(n\right)=\text{PAR}0*\prod_{\text{i}<n}\text{LIC}\left(n\right)$$ (36)

## Results

### Effective stem position measurement

**Fig 6:**
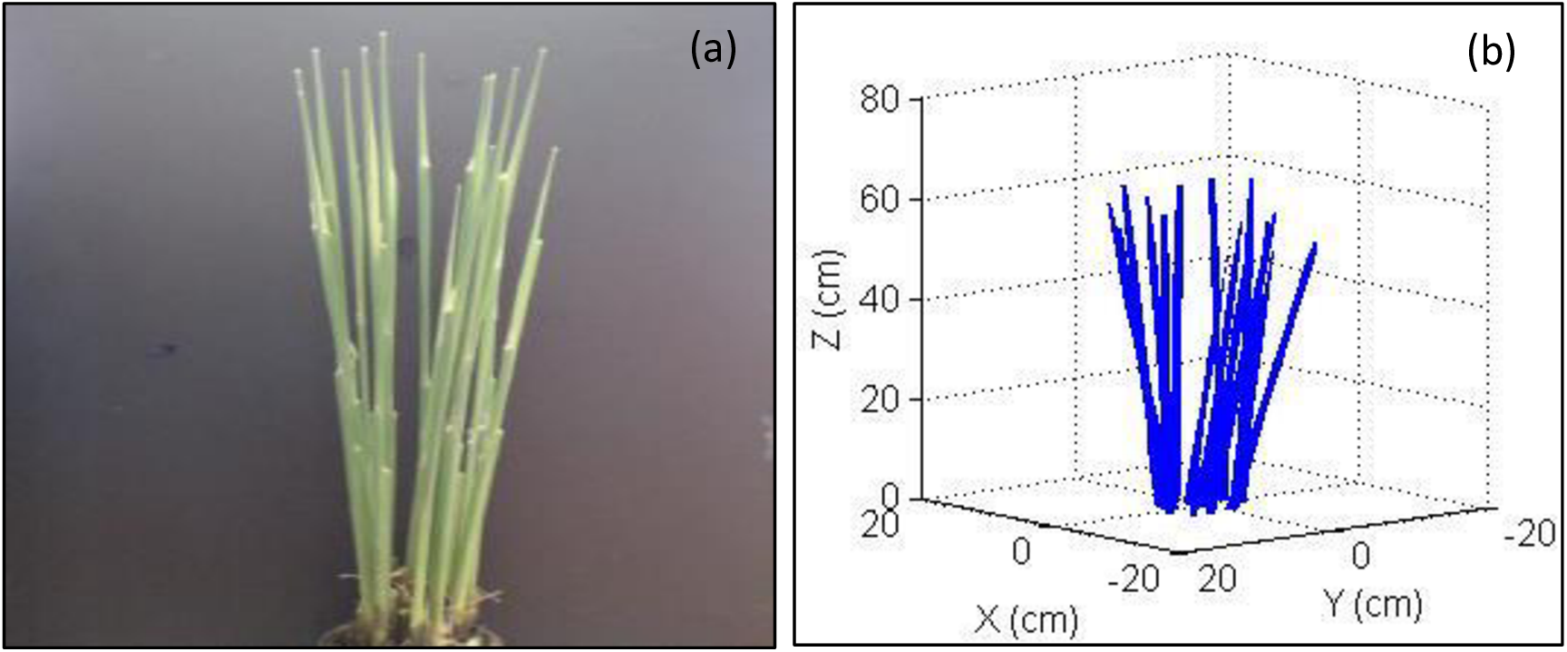
Stem image of a rice hill of Sample S6. The (**a**) presents the original image and (**b**) illustrates the 3D stem. The 3D stem was regenerated with the axis of the stem fitted by five measured points and presented as a cylinder determined by measured radius.

To test the accuracy of the measurement of the stem spatial position, the actual and simulated images of the plant stem were compared for Sample S6. The spatial position and radius at five points on the surface of each stem were measured with the CC tool. The five points on the axis of each stem were calculated with the measured five coordinates and radius. Fig 6B, for the 3D stem, was regenerated by fitting the stem axis with the calculated axis coordinates of the five points and the radius represented by the cylinder. For this, Fig 6B was visually comparable to its original image (Fig 6A). For a given stem, its digital stem axis was derived from the calculated stem axis coordinates of the five points. However, it could also be generated with the data of two points (bottom and top). Two types of stem axes did not have significant differences. Therefore, measurements at two points on the stem surface are sufficient to regenerate the digital spatial position of the stem.

### Validating the calculation of length, maximum width, and area of leaf

**Fig 7:**
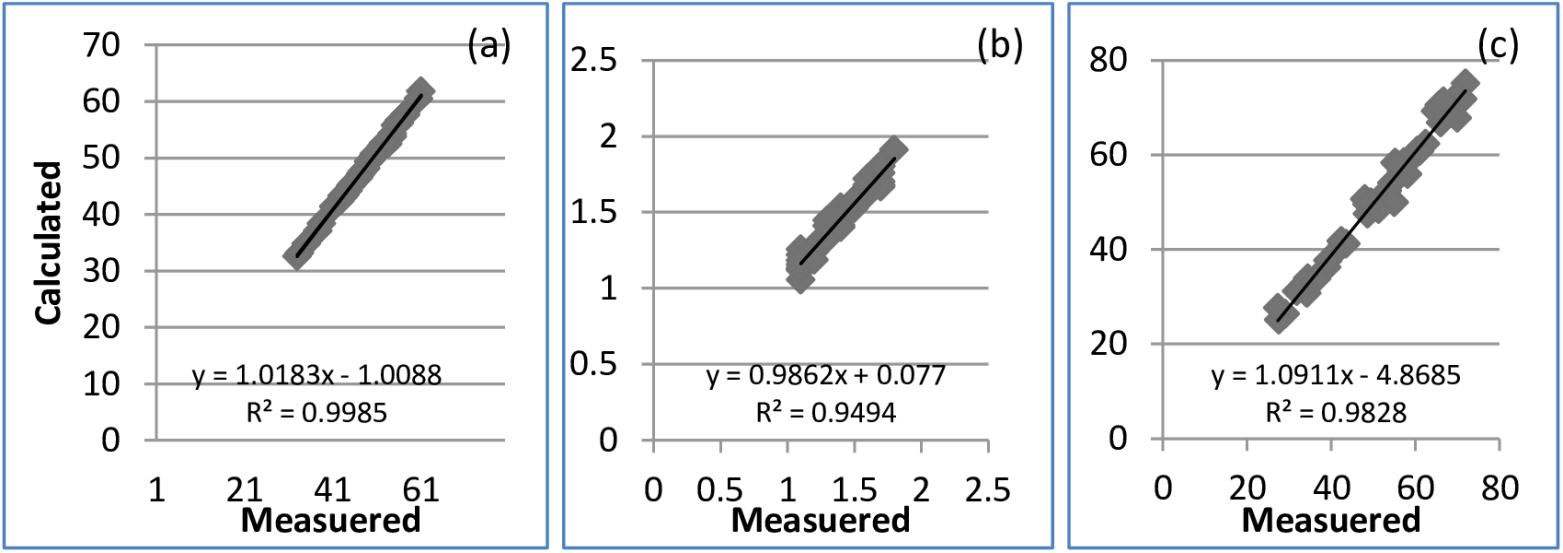
Comparison of the measured leaf length, maximum leaf width and leaf area with the calculated values using the photo method with measured directly of Sample S7, where (a) for comparision of leaf length (cm), (b) for comparision of leaf width (cm) and (c) comparision of leaf area (cm2).

A total of 40 leaves (5 groups from Sample S7) were used to validate the performance of the LIAE and the leaf image analysis. The data on length, maximum width, and area of each leaf generated through the image acquisition equipment and leaf image analysis were statistically compared with those measured using a ruler and the LI-COR LI-3100C leaf area meter (Fig 7A, 7B and 7C, respectively). The slope and correlation coefficients of the linear regression equations between the measured and calculated data were close to 1 (Fig 7). The data derived from the image analysis were reliable and were taken as the measured data.

### Validation of the calculation of base and dropping angles

**Fig 8:**
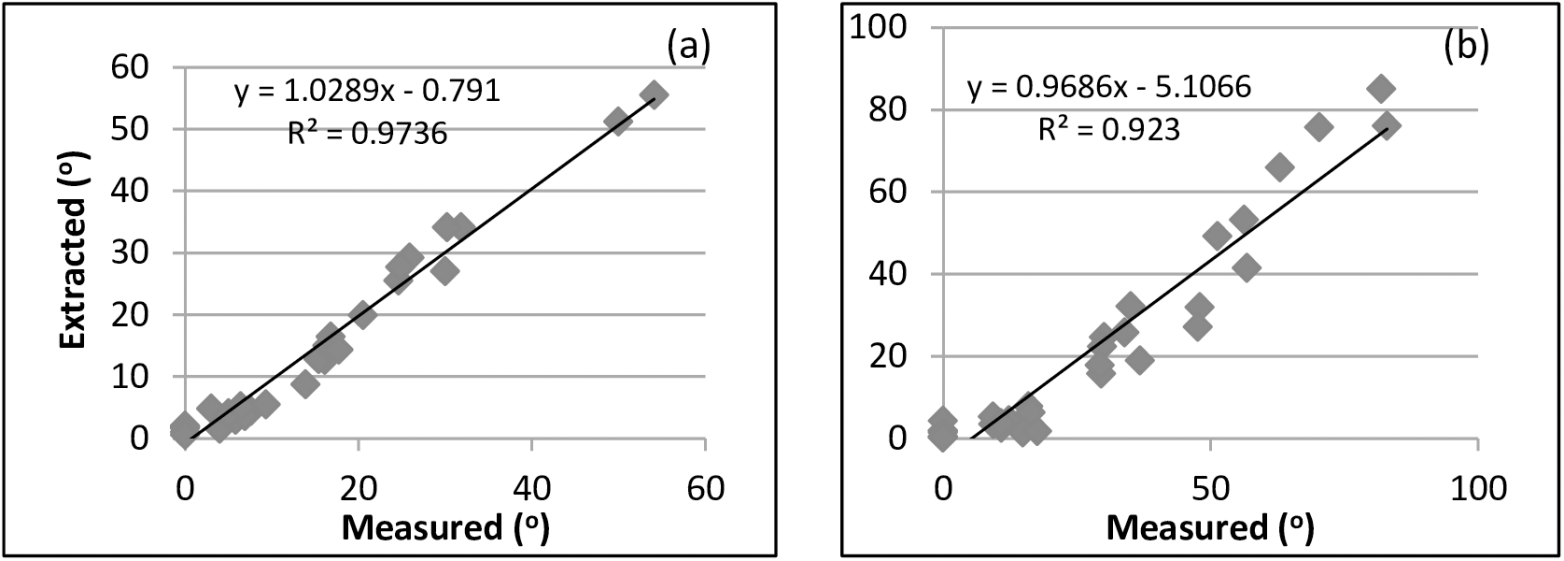
Comparison of leaf base angle and dropping angle generated using the photo method with measured ones directly from Sample S7, where (a) for comparision of leaf base angle and (b) for comparision of dropping angle.

To validate the performance of the TIAE and the tiller image analysis, data on the base and dropping angles of 33 leaf blades (7 tillers from sample S7) were extracted through the TIAE and tiller image analysis and also measured using a digital angle ruler. The measured base and dropping angles were very well represented by the extracted angles (Fig 8), with that the correlation and a slope of the linear regression between extracted and measuredvalues were approximately to1.

### Assessment of the reconstruction of the3D visual plant architecture

**Fig 9:**
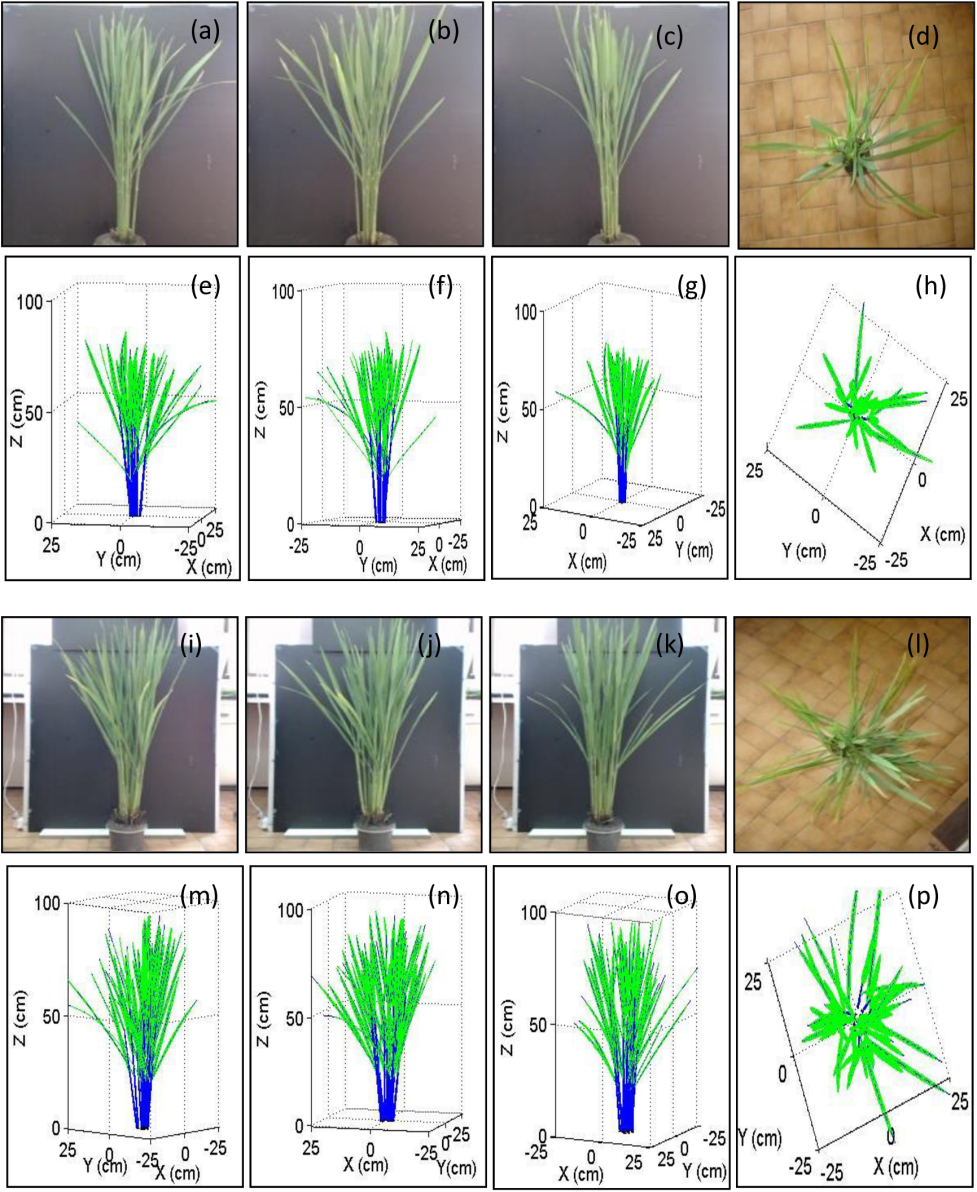
Assessment of the reconstruction of the 3D visual plant architecture. Pictures of the plant hills were taken before measurement (a to d for S4 and i to l for S5), and the virtual plants were reconstructed from the plant pictures (e to h for S4 and m to p for S5).

Samples S4 and S5 (two hills of the rice plant with trim-moldings) were used to assess the reconstruction of the rice plant canopy. Pictures of the plant hills from the side and top angles/views were taken before the measurements (Fig 9A to 9D and 9I to 9L). These pictures were analyzed, and the virtual plants were reconstructed using the method introduced in S2 File (Fig 9E to 9H and 9M to 9P). The virtual plants were comparable to the actual plants if the background was ignored.

### Assessment of the reconstruction model by architecture traits

To assess the technology system developed in this study, the light intensity (photosynthetic active radiation) and leaf area index were measured by a Sunscan SS1-UM-2.0 (Delta-T Devices Limited) at canopy layers in a interval of 20 cm from bottom to top, with three replications every 15 cm in each layer. The measurements were conducted at 13:30 on 10-March-2015.

**Fig 10:**
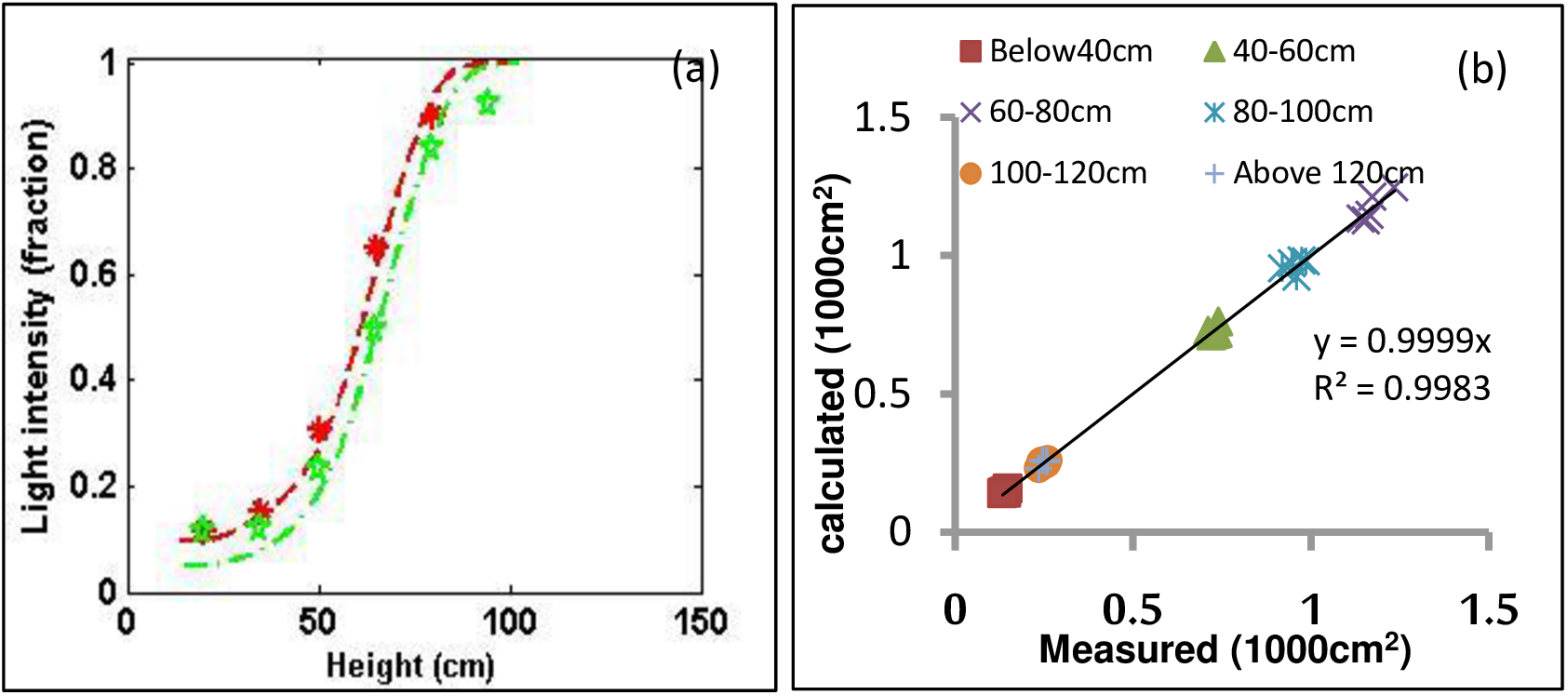
The measured light intensity fractions and leaf area in different canopy layers are in comparison with ones calculated from the digital architecture. In (**a**), the red and green dash lines represent calculated results of samples S1 and S2, while the red and green symbols are the measured values for S1 and S2, respectively. In the (**b**), symbols are the calculated against measured leaf areas at different canopy layers for Sample S3, and the line is 1:1 relationship line.

The measured and calculated light intensity fractions to the radiation at the top of the canopy (Fig 10A) has samll root mean square error which was 0.03 for sample S1, 0.04 for S2,. Moreover, the measured leaf area of the layers was represented very well by the calculated area (Fig 10B), with a root mean square error of 6.57, with correlation (i.e., R^2^ ≈ 1.0) and a slope of the linear regression between measured and calculated values were approximately to 1.

## Conclusions and discussion

This study focused on the measurement of rice plant architectures, the reconstruction and visualization of digital architectures, and trait extraction from the digital architectures. A technique system, consisting of the location-separation-measurement method (LSMM) for measurements, developing equipment for data acquisition and image capturing and software programs for image analysis, defining and reconstructing the data structure of the digital architecture, visualizing the 3D plant architecture, and using the virtual clipping method for trait extraction based on the digital architecture, was developed and validated.

This technique system is a low-cost and accurate system because it does not involve expensive devices, and the results demonstrated that the 3D visual architectures reconstructed using the technique system of this study were comparable to actual plant architectures, and the calculated leaf areas in the canopy layers and light interception at different depths of the canopy based on the digital plant architecture were in good agreement with the measured values. Moreover, the reconstruction of the 3D plant architecture based on directly obtained data is not the same as the L-system based on a statistical parameter [7].

This technique system achieves a high throughput. It can collect and extract plentiful information to satisfy plant phenotypic research and plant architecture research. In practical agricultural investigations, three people, working 10 hours, can measure 40–50 hills per day using this technique system. The system is more efficient than the digitizer reported by Zheng [18] because it processes the tiller and leaf as a whole; however, the digitizer can capture many points. On the other hand, given clean backgrounds, the image process of this technique system requires minimal manual intervention.

This study provided other highlights. For example, the data structure of the digital architecture should benefit the repeated use of data, the virtual clipping method should also be useful for the extraction of new indices of plant architectures, and the LSMM represents a feasible procedure for measuring sheltered plant architectures. However, this technique system suffers from certain defects. For example, the system has to operate indoors, the system cannot be operated in the field, and the measurement is destructive.

## Supporting information

S1 File Plant material. The detail information about the fields’ site, variety.
(DOCX)

S2 File Apparatus, Data structure, Reconstruction algorithm, Extraction algorithm and Processes for multi-dimension distributions of architecture traits. The details of apparatus (CC, TIAE, LIAE) and the operation; Algorithm of the reconstruction of the 3D visual geometrical structure of the rice canopy architecture in the field; Data structure of digital plant architecture; Algorithm of the reconstruction of the 3D visual geometrical structure of the rice canopy architecture in the field; Algorithm for trait extraction of canopy architecture using the virtual blade method; And processes involved in the virtual blade method for multi-dimension distributions of architecture traits
(DOCX)

S1 Fig The leaf image analysis for extracting the leaf shape profile data. Presentation of the four steps involved in the leaf image process: (a) original image, (b) binary image, (c) leaf image after pixel transformation, and (d) leaf shape curve after polynomial fitting.
(TIF)

S2 Fig The leaf image analysis for extracting the leaf venation profile data. Presentation of the four steps involved in the leaf image process: (a) original image, (b)binary image after gray processing, (c)tiller image after stem extraction, (d)tiller image after coordinate rotation, and (e)tiller image after equation fitting.
(TIF)

S3 Fig schematic diagram for virtual blade method. (a) virtual blade method with horizontal surfaces, (b) virtual blade method with cylindrical surfaces, 1: virtual blade sections, 2: the distance between two neighboring adjacent surfaces.
(TIF)

S1 Table Abbreviations. Abbreviations are listed with their full words and meaning.
(DOCX)

S1 Data Data collected for S4 and S5 digital plant architecture. There four type file in the zip file, SystemData is parameters of paratus, SSP_LCAA correspond to the special position of stem, leafvein correspond to data of leaf midrib curves, leafshape correspond to the data of leaf shape. 0: correspond to Sample S4, 1: correspond to Sample S5.
(ZIP)

S2 Data Data collected for S1 and S2 digital plant architecture. There four type file in the zip file, SystemData is parameters of paratus, SSP_LCAA correspond to the special position of stem, leafvein correspond to data of leaf midrib curves, leafshape correspond to the data of leaf shape. 101: correspond to neighbor 8 hills of Sample S1 (NSICRc222), 1: correspond to neighbor 8 hills of Sample S2 (NSICRc124H).
(ZIP)
