# Supplementary material for: A technique system for the measurement, reconstruction and character extraction of rice plant architecture
Source: PLoS One. 2017 May 30;12(5):e0177205. doi: 10.1371/journal.pone.0177205 (PMC5448746; doi:10.1371/journal.pone.0177205)
Supplement: S1 File — The detail information about the fields’ site, variety. (DOCX) [file pone.0177205.s001.docx]

## Plant material

Seven groups of samples (S1 to S7) were collected from two field sites (SS1 and SS2) for different purposes in this study. SS1 was located at the research farm of the International Rice Research Institute(IRRI), Los Baños, Philippines (21.25E, 14.18N, 21 m elevation), where two varieties of rice, NSICRc222 and NSICRc124H (Mes4), were sown on 19-Dec-2014 and transplanted into puddled lowland fields on 12-Jan-2015, with 25 hills per m^2^ (20×20 cm) and 2 plants per hill.SS2 was located at the research farm of Hunan Agricultural University, Liuyang, Hunan, China (113.51E, 28.23 N, 133.11 m elevation), where one variety, Shangyou63, was sown on 20-May-2012 and transplanted into padded lowland fields on 15-June-2012, with 19 hills per m^2^ (23×23 cm) and 1 plant per hill. The fields in both locations were managed with full water and nutrient supply. There was no permission needed to conduct an agricultural research in these two research farms because of their functional definition. Except for the local popular rice cultivars, water and ordinary nitrogen fertilizer used in these experiments, there were no endangered or protected species involved.

Samples S1 and S2 for NSICRc222 and NSICRc124H, respectively, were collected from 8 neighboring hills (two rows x four hills) in SS1 during the plant developmental stage(11-March-2015)and were used for the reconstruction of the 3D visual canopy architecture, trait extraction, and light distribution validation (light measurements in the field were conducted on 10-March-2015). Sample S3 for Shangyou63was collected from4 neighboring hills in SS2 on 14-Agust-2012 for the validation of the leaf area distribution along the vertical direction (z-axis).Samples S4 (NSICRc222, 1 hill) and S5 (NSICRc124H, 1 hill) were collected from SS1 on 13-March-2015 for the visual comparison between the reconstructed plant architecture and photos of the original architecture taken prior to measurement. SampleS6 (NSICRc124H, 1 hill) was collected from SS1 on 27-March-2015 for the assessment of the stem position measurement. Sample S7 (NSICRc124H, 2 hills) was collected from SS1 during the plant developmental stage (13-March-2015) and was used for validating the calculation of the leaf length, maximum width, and area and for the reconstruction of the leaf orientation.
