# Supplementary material for: A technique system for the measurement, reconstruction and character extraction of rice plant architecture
Source: PLoS One. 2017 May 30;12(5):e0177205. doi: 10.1371/journal.pone.0177205 (PMC5448746; doi:10.1371/journal.pone.0177205)
Supplement: S2 File — The details of apparatus (CC, TIAE, LIAE) and the operation; Algorithm of the reconstruction of the 3D visual geometrical structure of the rice canopy architecture in the field; Data structure of digital plant architecture; Algorithm of the reconstruction of the 3D visual geometrical structure of the rice canopy architecture in the field; Algorithm for trait extraction of canopy architecture using the virtual blade method; And processes involved in the virtual blade method for multi-dimension distributions of architecture traits (DOCX) [file pone.0177205.s002.docx]

## Apparatus

### Cylindrical Coordinatograph

The CC tool consisted of a measuring console, rotatable round table, height gauge, wedge-shaped metric ruler, and digital angle ruler (Fig. 2). The rotatable round table had a horizontal angle scale from 0° to 360°.A smooth, hard, square board, which was smaller than the round table, was set between the console and the round table. A circular shaft passing through the smooth, hard, square board and round turntable was settled in the console to ensure that the round table rotated conveniently and smoothly. The height gauge with seven fixed horizontal bars stood vertically above the console to measure the position of the stem.

The CC tool was used to determine the spatial position of a point on the stem and the azimuth of a leaf through the following steps:

1. The hill of the rice plant was set on the center of the round turntable with the marked north direction of the hill aligned to zero degrees on the round turntable.
2. The tip of the wedge-shaped metric ruler was made to touch a stem of the hill by rotating the round turn table and sliding the ruler on a bar and against a side face of the height gauge (Fig. 2a).
3. The following data were then measured: the distance from the touch point to the edge of the height gauge (*d*), the angle scale directing to the height gauge (α), and the distance from the touch point to the soil plane(*h*), which is the height of the bar above the round turn table minus the distance from the soil plane to the round table. The coordinates of the touch point on the stem were(*R-d*,α, *h*), which were relative to the cylindrical coordinate system, with the origin at the center point of the round turntable, the longitudinal axis up straight, the polar axis being zero scale on the round turntable, and *R* being the distance from the longitudinal axis to the edge of the height gauge.

This tool was also used to determine the azimuth of the leaf on the stem through the following steps:

1. The digital ruler was placed on one of the horizontal bars, moved until it touched the stem, and adjusted on another leg parallel to the vertical section of the leaf midrib (Fig. 2b).
2. The angle of the digital ruler$(\theta_{1})$ and the angle shown on the round turntable ($\theta_{2}$), which is directed toward the height gauge, were recorded. The azimuth of a leaf midrib was computed as $\theta=\theta_{2}+\theta_{1}$.


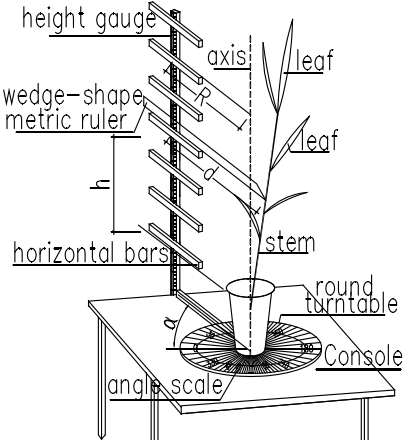

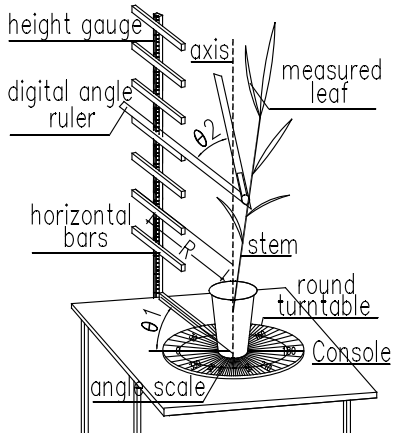


**(a)**

**(b)**

Fig 1.Schematic diagram of the structure and operation of the CC tool

### Tiller image acquisition equipment

The TIAE was designed to capture an image of a single tiller as a reference for extracting the curvature of the leaf blade using the software developed in this study (see section 2.5.2). It is composed of a frame, background plate, camera, camera mounting bar, validation paper, and stem anchor needle (Fig. 3a). The background platewasa black board standing upright; the stem anchor needle was a wooden block with an iron hairpin used to hold and fix the tiller on the background plate; the camera was connected to a computer, placed facing the background plate, and mounted on the standing bar attached to the frame; and the validation paper was a sheet of white paper with black grid points.

An image of the tiller was taken, with special attention given to the following:

1. The height of the camera and the distance to the background plate should be adjusted to ensure that the whole tiller is captured in the photo.
2. The tiller should stand against the background plate, and all the leaves of the tiller should be close to the background plate in their natural posture by rotating the sheath.
3. The captured image should be stored in the computer.

Steps 2-3 were repeated if there were additional leaves to be processed. Before or after taking the photo, the validation paper was placed in front of the background plate to provide reference on the real coordinates (Fig. 4).


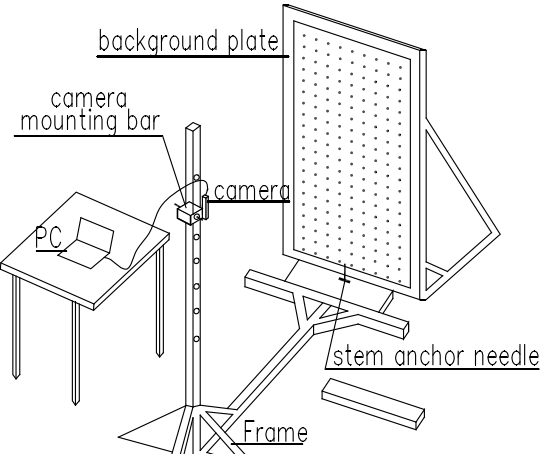

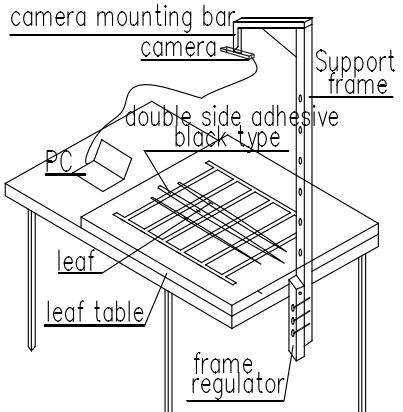


**(a)**

**(b)**

Fig 2.Schematic diagram of the structure of the tiller image acquisition equipment (a) and the leaf image acquisition equipment (b)

### Leaf image acquisition equipment

The LIAE was designed to take photos of leaves as references for extracting leaf shape data through the software developed in this study. The equipment consisted of a support frame, frame regulator, leaf table, camera, camera mounting bar, validation paper, and black double-sided adhesive tape. The leaf table was a horizontal black desk. The camera was connected to a computer and mounted above the desk using support frames that stood upright and were fixed in position by the frame regulator using the isometric holes (Fig. 3b).

An image of the leaves of a tiller was taken, with special attention given to the following:

1. The height of the camera should be adjusted with the frame regulator to ensure that all leaves will be captured in the photo.
2. The leaves should be completely expanded and carefully pasted on the leaf table using the double-sided adhesive tape.
3. All leaves of the tiller should be arranged on the table in the order of phyllotaxy.
4. All photos taken should be stored in the computer for future analysis.

Steps3-4 were repeated if there were additional leaves to be processed. Before or after taking photos, the validation paper was placed on the leaf table to provide reference on the real coordinates.


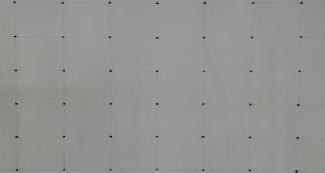

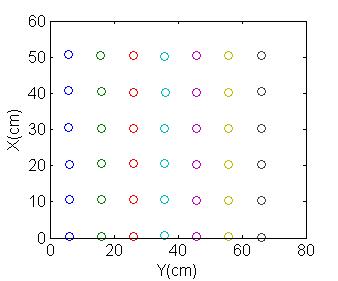


(b)

(a)

Fig 3.Schematic diagram of the validation paper

### LSMM for a multiple hill community

(1) Hills were sampled into a pot with soil, and their field locations were recorded using their cropping column and row number and direction. Through this process, the canopy was maintained at its original posture in the field, with the rice plant upright inside the pot with its north direction in the field.

(2) The positions of the stem and the azimuth of the leaf of a tiller were measured using CC. The tiller was then separated from the hill to prepare it to be photographed.

(3) The tiller image was taken using TIAE; then, the leaves were detached from the tiller to prepare them to be photographed.

(4) The leaf image was taken using LIAE.

(5) Steps3-4 were repeated for all tillers of a hill, and steps2-4 were followed for all hills.

### Data structure of digital plant architecture

For Field: planting space, sampled plant number (pm), additional information about environmental conditions and management intervention etc.

For Hill: hill position in the field

For Tiller: stem spatial position, length, and radius

For Leaf: leaf node, azimuth, shape, and venation curve

### Algorithm of the reconstruction of the 3D visual geometrical structure of the rice canopy architecture in the field

For Hill in Field

For Tiller in Hill

For Stem in Tiller

***stem spatial position = stem spatial position + (Hill position – 1)* Planting space;***

Restructure Stem with ***stem spatial position*** and ***Stem radius***

For Leaf in Stem

***Leaf midrib curve = Leaf midrib curve + (Hill position – 1)* Planting space;***

***Leaf shape = Leaf shape + (Hill position – 1)* Planting space;***

Restructure Leaf with ***Leaf midrib curve*** and ***Leaf shape***

End(Stem)

End(Tiller)

End(Hill)

End(Field)

### Algorithm for trait extraction of canopy architecture using the virtual blade method

For Hill in Field

For Tiller in Hill

For Stem in Tiller

For Leaf in Stem

For Fragments in Leaf

Calculate the traits of the small fragment;

Calculate the interval to which the small fragment belongs;

Make a summary of the traits of each small fragment that belongs to interval (voxel);

End (Leaf)

End (Stem)

End (Tiller)

End (Hill)

End (Field)

### Processes involved in the virtual blade method for multi-dimension distributions of architecture traits

1) Reconstruct the 3D digital plant architecture from the collected data.

2) Split the space of the canopy into m×n or m×n×lsub-spaces named voxels.

3) Split the leaves into small fragments and compute the traits (leaf area, azimuth and inclination) of each small fragment.

4) Summarize the traits of each small fragment in voxels.
