# Supplementary figures and images for: A technique system for the measurement, reconstruction and character extraction of rice plant architecture
Source: PLoS One. 2017 May 30;12(5):e0177205. doi: 10.1371/journal.pone.0177205 (PMC5448746; doi:10.1371/journal.pone.0177205)

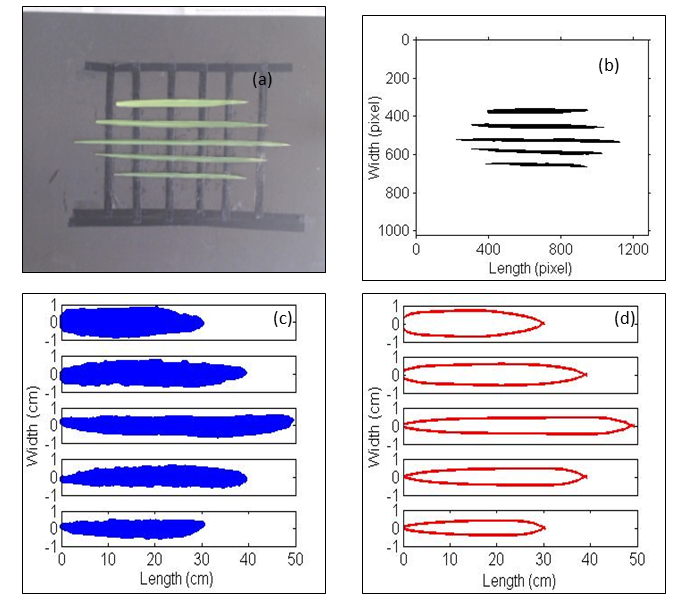

Supplement: S1 Fig — Presentation of the four steps involved in the leaf image process: (a) original image, (b) binary image, (c) leaf image after pixel transformation, and (d) leaf shape curve after polynomial fitting. (TIF) [file pone.0177205.s003.tif]

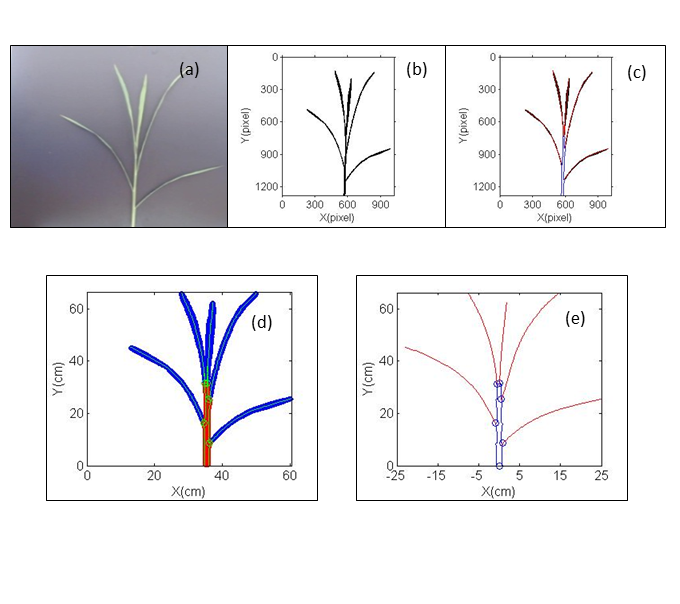

Supplement: S2 Fig — Presentation of the four steps involved in the leaf image process: (a) original image, (b)binary image after gray processing, (c)tiller image after stem extraction, (d)tiller image after coordinate rotation, and (e)tiller image after equation fitting. (TIF) [file pone.0177205.s004.tif]

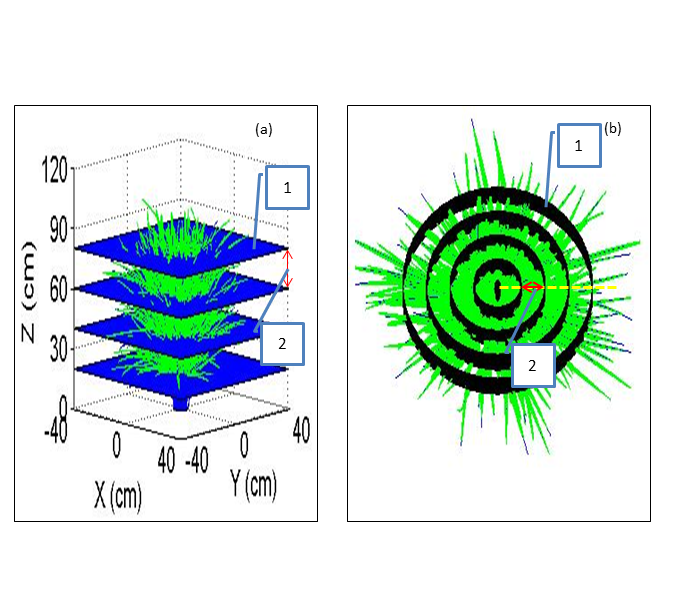

Supplement: S3 Fig — (a) virtual blade method with horizontal surfaces, (b) virtual blade method with cylindrical surfaces, 1: virtual blade sections, 2: the distance between two neighboring adjacent surfaces. (TIF) [file pone.0177205.s005.tif]
