# Supplementary material for: A technique system for the measurement, reconstruction and character extraction of rice plant architecture
Source: PLoS One. 2017 May 30;12(5):e0177205. doi: 10.1371/journal.pone.0177205 (PMC5448746; doi:10.1371/journal.pone.0177205)
Supplement: S1 Table — Abbreviations are listed with their full words and meaning. (DOCX) [file pone.0177205.s006.docx]

| **Abbreviation** | **Full Words** | **Meaning** |
| --- | --- | --- |
| CC | Cylindrical Coordinatograph | An apparatus to measure spatial position and leaf azimuth |
| LSMM | location-separation-measurement method | a method to collection plant architectural data |
| TIAE | tiller image acquisition equipment | An apparatus to acquire tiller image |
| LIAE | leaf image acquisition equipment | An apparatus to acquire leaf image |
| *L_l_* | leaf length | leaf length |
| *L_mw_* | the maximum leaf width | the maximum leaf width |
| *Lc* | the dataset of the real coordinates of the leaves for defining the leaf curve | the dataset of the real coordinates of the leaves for defining the leaf curve |
| *S* | The dataset of the real coordinates of the stem for defining the stem shape | The dataset of the real coordinates of the stem for defining the stem shape |
| *S_l_* | the length of the stem | the length of the stem |
| *S_r_* | the radius of the stem | the radius of the stem |
| LN | the position of the leaf node | the position of the leaf node |
| LM | the point set of the midrib of a stem | the point set of the midrib of a stem |
| *LMO* | the point dataset of the midrib | the point dataset of the midrib |
| (*rs*, *ps*) | the row and plant space | the row and plant space |
| (*rn, cn*) | **hill position** | The **hill position** in the field is represented by the row and column numbers in the sampling block |
| *D_ns_* | the distance between two adjacent surfaces | the distance between two adjacent surfaces along the z-axis |
| *farea* | Area of fragment | Area of fragment |
| *finterval* | The order number of intervals for the fragments | The order number of intervals for the fragments |
| *Area(n)* | The leaf area in interval *n* | The leaf area in interval *n* |
| *Areadis(n)* | the leaf area density distribution | the leaf area density distribution |
| *Areapdis(n)* | the leaf area probability distribution | the leaf area probability distribution |
| *Azimuthdis(n)* | the leaf azimuth distribution | the leaf azimuth distribution |
| *incination(n)* | the leaf inclination distribution | the leaf inclination distribution |
| *PSAREA(n)* | The total projection area on the plane perpendicular to the sun | The total projection area on the plane perpendicular to the sun |
| *LIC(n)* | The light interception coefficient of the interval *n* | The light interception coefficient of the interval *n* |
| *LID(n)* | the light relative density distribution | the light relative density distribution |
